# Animal-Free Human Whole Blood Sepsis Model to Study Changes in Innate Immunity

**DOI:** 10.3389/fimmu.2020.571992

**Published:** 2020-10-14

**Authors:** David Alexander Christian Messerer, Laura Vidoni, Maike Erber, Alexander Elias Paul Stratmann, Jonas Martin Bauer, Christian Karl Braun, Stefan Hug, Anna Adler, Kristina Nilsson Ekdahl, Bo Nilsson, Eberhard Barth, Peter Radermacher, Markus Huber-Lang

**Affiliations:** ^1^Institute of Clinical and Experimental Trauma Immunology, University Hospital of Ulm, Ulm, Germany; ^2^Department of Anesthesiology and Intensive Care Medicine, University Hospital of Ulm, Ulm, Germany; ^3^Rudbeck Laboratory, Department of Immunology, Genetics and Pathology, Uppsala, Sweden; ^4^Centre of Biomaterials Chemistry, Linnaeus University, Kalmar, Sweden; ^5^Institute for Anesthesiologic Pathophysiology and Process Engineering, Ulm University, Ulm, Germany

**Keywords:** inflammation, neutrophil granulocytes, lipopolysaccharide (LPS), blood physiology, sepsis, ex vivo whole blood model, principles of the 3Rs

## Abstract

Studying innate immunity in humans is crucial for understanding its role in the pathophysiology of systemic inflammation, particularly in the complex setting of sepsis. Therefore, we standardized a step-by-step process from the venipuncture to the transfer in a human model system, while closely monitoring the inflammatory response for up to three hours. We designed an animal-free, human whole blood sepsis model using a commercially available, simple to use, tubing system. First, we analyzed routine clinical parameters, including cell count and blood gas analysis. Second, we demonstrated that extracellular activation markers (e.g., CD11b and CD62l) as well as intracellular metabolic (intracellular pH) and functional (generation of radical oxygen species) features remained stable after incubation in the whole blood model. Third, we mimicked systemic inflammation during early sepsis by exposure of whole blood to pathogen-associated molecular patterns. Stimulation with lipopolysaccharide revealed the capability of the model system to evoke a sepsis-like inflammatory phenotype of innate immunity. In summary, the presented model serves as a convenient, economic, and reliable platform to study innate immunity in human whole blood, which may yield clinically important insights.

## Introduction

Innate immunity plays a vital role as the vanguard against numerous harmful carriers of damage- and pathogen associated molecular patterns (DAMPs and PAMPs, respectively). An appropriate inflammatory response efficiently clears pathogens and initiates subsequent healing processes. However, an excessive and dysregulated inflammation is a matter of both increasing interest and concern, particularly during trauma (1) and sepsis (2). A recent study estimated the annual global incidence of sepsis at approximately 50 million (3). Moreover, the latest consensus definition of sepsis underscored the importance of appropriate regulated (innate) immunity in the successful clearance of pathogens (2).

To study the complex innate immune response to PAMP exposure, various small or large, simple or complex animal models are used (4–6). However, ethical considerations, including the aspect of replacement within the 3R-principles (7), possible limitations in translation into real-world patients (4, 5), and the expense of personal and material resources, may limit the validity and attractiveness of animal-based models. In addition, whole blood samples or tissue specimens of patients suffering from sepsis can only be obtained at the cost of additional burden and risk to the patients. Moreover, the samples originate from different etiological, microbiological, and genetic backgrounds, and thus cannot be standardized precisely. For example, the exact exposure time and the amount of pathogens within the blood during sepsis cannot be determined with certainty. Therefore, as another alternative, injection of immune-stimulants, including lipopolysaccharide (LPS), has been performed and investigated in healthy human volunteers (8–10). On the one hand, this approach provides the intriguing opportunity to study the immunological response directly in human organisms. On the other hand, obvious ethical considerations limit the possibilities of this method, for example, constraining the severity of the induced inflammation or being restricted to young and healthy individuals (9). However, because excessive inflammation during sepsis remains a major clinical and scientific challenge, there is an unabated research need to elucidate the underlying immunological pathomechanisms, for example, by utilizing human whole blood in a standardized and reliable manner.

Exposure of blood to DAMPs, PAMPs, and/or defined pathogens outside the human body surmounts several limitations indicated above. Notwithstanding, there are some cellular and humoral components in the blood that become rapidly activated when losing their contact with intact endothelium, which under homeostatic conditions acts as an anti-inflammatory, anti-adhesive, and anti-coagulatory regulator. In particular, platelets and serine cascades are controlled by an intact endothelium (11). To address the issue of “hemoincompatibility” of artificial materials, whole blood is frequently completely anticoagulated, for example, either by heparin-based anticoagulants or cation-chelators, including citrate, both of which can alter the ability of humoral and cellular immunity to adequately respond to stimulation.

To prevent stimulation after venipuncture *ex vivo*, a variety of models have been previously proposed of whole blood being supplemented with an anticoagulant and/or being encased in a specially coated system. Human whole blood models of coated circuits or whole blood cultures in combination with defined anticoagulation, for example, heparin or hirudin, have been widely used and characterized in recent years (12–18). In general, these models address a specific focus and produced the respective specific answers. For example, they were used to elucidate the interaction of whole blood stimulated with various bacteria, revealing the significance of the complement factors 3a and 5a (C3a, C5a) as well as CD14 for the crosstalk of pathogens and leukocytes (12–15).

However, to our knowledge, the description of hitherto existing models focused on the characterization of humoral immunity and other protease systems such as the coagulation cascade, without elucidating the impact of exposition to an *ex vivo* circuit to general (patho-)physiological and metabolic responses in association with cellular innate immunity. For example, global blood parameters (e.g. pH and glucose concentration) have frequently not been reported, making it difficult to interpret the presented data and to transfer them into the physiological *in vivo* context of the human body (12–14, 16, 18–21). There is also a concomitant research gap with regards to a comprehensive study of blood physiology *ex vivo* while synchronically assessing important immunological functions and activation markers.

Here, we 1) standardized the handling of an easy-to-use whole blood model, 2) determined a myriad of global blood parameters with a comprehensive focus on extra- and intracellular parameters of innate immunity, and 3) mimicked septic conditions and subsequent systemic inflammation in blood in an animal-free research setup by stimulation with LPS.

## Methods

### Blood Sampling

Healthy human volunteers of both genders aged 21–30 years served as blood donors. All experiments were conducted in accordance with the declaration of Helsinki (22). Following ethical approval (number 459/18, Local Independent Ethics Committee of the University of Ulm) and written informed consent, blood was drawn by peripheral venipuncture in accordance with the guidelines of the World Health Organization (23). In detail, blood stasis was limited to a maximum of 30 s before puncture and the first 2–3 ml were immediately discarded. The blood was collected in neutral monovettes (Sarstedt, Nürnbrecht, Germany), which had been supplemented with heparin (B. Braun Melsungen AG, Melsungen, Germany) and either LPS (Sigma Aldrich, Steinheim, Germany) or phosphate-buffered saline (PBS^++^, as control), respectively. In total, 9 ml blood was collected per tube, with a final concentration of 0.5 IU/ml of heparin and when present, 100 ng/ml of LPS.

### Whole Blood Model

Immediately after sampling, the blood was transferred carefully using 10 ml tips (Eppendorf, Hamburg, Germany) into the following described system. The whole blood model consisted of a heparin coated tubing system (Cortiva, #M999413C, Medtronic, Meerbusch, Germany), being cut into approximately 33 cm long pieces. Both ends of the tube were added to a circuit using a similarly coated connector (Cortiva, #CB4629, Medtronic), leaving an air bubble of approximately 1.5 ml inside the system. The loops were attached to a spinning wheel (Snijders Labs, Tilburg, Netherlands) rotating at 5 rpm, causing the air bubble to generate a continuous circulation of the blood. As described previously, the combination of heparin-coating and continuous circulation mediated by a small air bubble allows incubating whole blood with a low-dose of heparin (24). Another option would be the application of higher doses of heparin, however, this interferes with important immunological systems such as the complement system (24) or the leukocytes (25). The authors actively decided against to use a mechanic pump, as this causes mechanical stress, that among other consequences results in hemolysis and platelet activation (26).

The system was incubated for 0 (depicted as “0^−^” indicating no contact with the tubing system), 10, 60, or 180 min as indicated in an incubator at 37°C without additional CO_2_. Following the incubation period, the tubing loops were cut open. Initially, 95 µl blood were directly drawn into dry-heparin anticoagulated glass capillary tubes (Radiometer GmbH, Krefeld, Germany). Subsequently, 1 ml of the blood was transferred into a heparin anticoagulated tube (Sarstedt) for the analysis of phagocytotic activity and radical generation. The remaining blood was transferred into citrate anticoagulated monovettes (Sarstedt) for the analysis of all other parameters. Sodium, potassium, ionized calcium, lactate, glucose, and blood pH were determined using a standard blood gas analyzer (ABL 800 Flex, Radiometer GmbH). To calculate the difference in the amount of lactate generated or the consumption of glucose, the respective value after 60 min with or without incubation with LPS was subtracted from the corresponding baseline (0^−^). Differential blood count and global coagulation parameters (activated partial thromboplastin time, aPTT, and international normal ratio (INR)) were determined using a standard hematology (Sysmex CN 2000, Sysmex, Kobe, Japan) and coagulation (BCS XP, Siemens, Marburg, Germany) analyzer, respectively, each according to the manufacturer’s standard protocol.

### Flow Cytometry

For antibody staining, 10 µl citrate anticoagulated blood were added to 40 µl PBS^++^ adjusted to pH 7.3 and stimulated with 100 ng/ml C5a (Complement Technology, Tyler, Texas, USA) or PBS as control for 15 min in a water bath at 37°C. Subsequently, the cells were stained as indicated with anti-CD11b (APC, dilution 1:82 #101212, BioLegend, San Diego, California, USA), anti-CD14 (APC-Cy7, dilution 1:200 #301820, BioLegend), anti-CD62l (PE, dilution 1:33 #304806, BioLegend), anti-CD88 (APC, dilution 1:250 #344310, BioLegend), or corresponding isotype controls (all from BioLegend), respectively, for 15 min at room temperature. All markers were assessed by monoclonal mouse anti-human antibodies. Cellular viability was analyzed by identifying necrotic cells with the Zombie Violet Fixable Viability Kit (dilution 1:4000, #423114, BioLegend) and apoptotic cells using Apotracker Green (final concentration 200 nM, #427402, BioLegend).

The generation of radical oxygen species (ROS) was determined by staining 40 µl heparin anticoagulated blood with 0.29 mM (100 µg/ml) dihydrorhodamine 123 (DHR, Santa Cruz Biotechnology, Dallas, Texas, USA). Phagocytosis was analyzed using fluorescent microspheres (Fluoresbrite™ Carboxylate YG 0.75 Microspheres, Polysciences, Inc., Warrington, Pennsylvania, USA). The microspheres were dissolved 1:20 in PBS^++^ followed by a washing procedure (3× at 1000 g for 5 min). Of this microsphere solution, 10 µl was added onto 100 µl heparin anticoagulated whole blood. Whole blood stained with either DHR or incubated with microspheres was incubated for 20 and 30 min, respectively, at 37°C in the dark. In all experiments, after stimulation and staining of whole blood, the erythrocytes were lysed and the leukocytes fixed by filling up the sample volume to 1 ml with 1× BD FACS lysing solution™ (BD Biosciences, San Jose, California, USA) for 30 min at room temperature in the dark. Following centrifugation of the samples for 5 min at 340 g, the specimens were resuspended in 100 µl PBS + 0.1% bovine serum albumin and stored at 4°C until further analysis (normally within 1 h).

For the analysis of living granulocytes and plasma, citrate anticoagulated whole blood was centrifuged for 10 min at 400 g at room temperature. The supernatant plasma was carefully removed from the hematocrit and stored at −80°C until further use for enzyme-linked immunosorbent assay (ELISA) analysis. The remaining blood cells were subjected to dextran sedimentation followed by hypotonic lysis of the erythrocytes. Forward scatter (FSC) area was used as a surrogate for cellular shape (27). The intracellular pH was determined using the fluorescent dye SNARF (Thermo Fisher Scientific, Waltheim, Massachusetts, USA) with nigericin-based calibration curves as described previously (28).

For all flow cytometry experiments, neutrophils and monocytes were gated based on their forward and sideward scatter (SSC) area properties. Doublets were removed by plotting forward scatter area versus height. Spillover between fluorescence channels was corrected by a compensation matrix. For all antigens, appropriate isotype controls (see figures) and single-staining controls were performed (data not shown). For all experiments, a minimum of 300 monocytes and 1000 neutrophils were recorded using a FACS Canto II (BD Biosciences).

### Determination of Platelet-Neutrophil-Complexes

Platelet-neutrophil-complexes (PNC) were analyzed by light microscopy (29). In brief, 100 µl citrate anticoagulated blood was diluted with 100 µl PBS^++^. Blood smears were stained with the “Hemacolor^®^ Rapid staining of blood smear - staining set for microscopy” (Merck KGaA, Darmstadt, Germany). A minimum of 50 neutrophils per specimen were analyzed by two independent and blinded individuals. Each neutrophil with at least one thrombocyte in direct proximity was counted as a PNC.

### ELISA

Determination of plasma levels of C3a, matrix metallopeptidase 9 (MMP9), interleukin 6 (Il6), and interleukin 8 (Il8) was conducted by standard ELISA as indicated by the manufacturer (MMP9: R&D Systems, Minneapolis, Minnesota, USA; Il6: BD Biosciences; Il8: R&D Systems; C3a: Quidel Corporation, San Diego, California, USA).

### Data Analysis and Statistics

All data is presented as median with error bars indicating the interquartile range (IQR). When informative (e.g. to assess donor heterogeneity in cellular immunity), additional scatter plots are presented as overlay. The sampling strategy consisted of an initial experiment series with n = 3 for all time points (0^−^, 10 min, 60 min, 180 min, 60 min with LPS, 180 min with LPS) focusing on blood gas analysis, whole blood count, and humoral inflammation. Because blood glucose, pH, and lactate level became unphysiological beyond 1 h and previous literature indicated that the neutrophil phenotype as measured by CD11b/CD62l expression reached its final alteration after exposure to LPS for 1 h (30), cellular immunity was analyzed for this time point from at least five independent specimen. Data analysis was performed with licensed versions of Microsoft Excel 2019 (Microsoft, Redmond, Washington, USA) and GraphPad Prism 8 (GraphPad Software Inc, San Diego, California, USA). In statistical testing, data was considering as paired and non-normally distributed: For comparison of two groups, a Wilcoxon matched-pairs signed rank test was conducted (**Figures 4**, **5**, comparison of control vs. C5a within the respective condition). Multiple-group comparison was performed by the Friedman test for paired data (**Figures 4**–**6A, B**, comparison of 0^−^ vs. Ctrl vs. LPS) or Kruskal-Wallis test for unpaired data (**Figures 6C, D**, comparison of 0^−^ vs. Ctrl vs. LPS), respectively, followed by Dunns’ multiple comparison test. A *p*-value <0.05 was considered to be significant and marked by * or **, indicating <0.05 or 0.01, respectively.

## Results

### Stability of Global Physiological Parameters in the *Ex Vivo* Whole Blood Model During the First Hour

**Figure 1:**
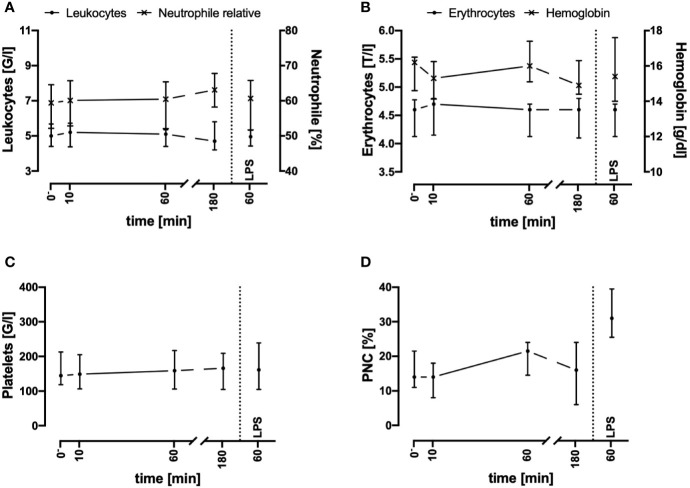
Cell counts of leukocytes **(A)**, erythrocytes **(B)**, platelets **(C)**, and platelet-neutrophil complexes (PNC) **(D)** before and after contact of blood with the tubing system. Cell counts and PNC formation remained stable during the first 3 h after exposure of whole blood in the ex vivo model. LPS (100 ng/ml) was used as a potent PAMP stimulus. n = 3. Results are presented as median with error bars indicating the interquartile range.

In the present whole blood model, the leukocyte and erythrocyte cell counts remained stable for up to 3 h after circulation in comparison to baseline values (directly after venipuncture, **Figures 1A, B**). In accordance, the amount of necrotic and/or apoptotic cells for neutrophils, monocytes, and lymphocytes was determined to be <2% (n = 3, data not shown). Furthermore, the activity of hemostasis and the coagulation system was monitored: platelet count (**Figure 1C**) and the formation of PNCs (**Figure 1D**) indicated no initiation of cellular coagulation. As expected, after the addition of 0.5 IU heparin/ml and incubation for 60 min, humoral coagulation was inhibited as indication by an INR of 2.1 (1.8; 2.3, n = 4) and an aPTT of >180 s in all four analyzed samples (data not shown). No visible clot formation was detected in any of the analyzed specimens.

**Figure 2:**
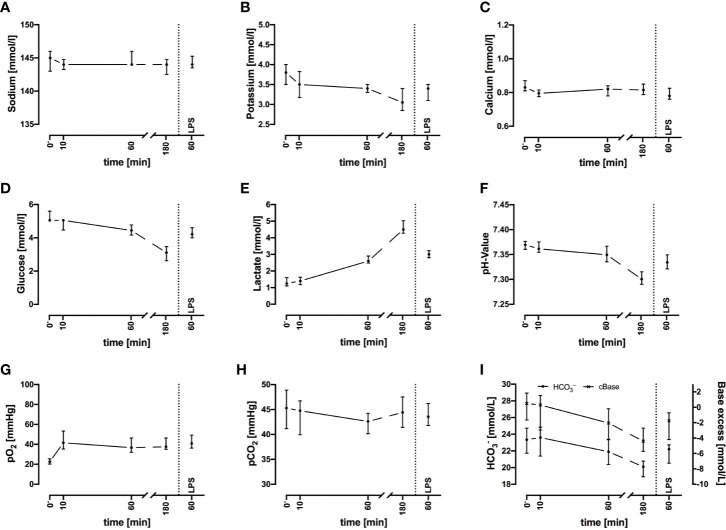
Sodium **(A)**, potassium **(B)**, ionized calcium **(C)**, glucose **(D)**, lactate **(E)**, pH **(F)**, oxygen partial pressure (pO_2_) **(G)**, carbon dioxide partial pressure (pCO_2_) **(H)**, and acid-base balance **(I)** before and after exposure of whole blood to the circuit system. Plasma parameters remain widely stable during the first 60 min but show deviations after 3 h. LPS (100 ng/ml) served as PAMP exposure. n = 3 for 10 min and 180 min, n = 10 for all other time points. Results are presented as median with error bars indicating interquartile range.

Electrolyte concentrations including sodium, potassium, and ionized calcium were stable throughout the 3-h period of interest (**Figures 2A–C**). Because the potassium levels did not increase and the red blood cell count and hemoglobin did not decrease, no considerable hemolysis had occurred in the system.

Regarding metabolic changes, there was an expected glucose consumption over time. The glucose concentration (**Figure 2D**) decreased within the first hour of incubation with a median of –16% (–12%; –17%, n = 10), with a glucose consumption rate of 0.80 mmol/L/h (0.60; 0.92, n = 10). The lactate (**Figure 2E**) concentration almost doubled (+94%, 88%; 126%) within the first hour, accounting for a generation rate of lactate of 1.4 mmol/L/h (1.1; 1.6, n = 10). The pH (**Figure 2F**) and acid-base balance (**Figure 2I**) changed accordingly. Partial pressure of oxygen (pO_2_, **Figure 2G**) increased slightly within the first 10 min, while partial pressure of carbon dioxide (pCO_2_, **Figure 2H**) remained stable. Both parameters indicated that the tubing system was airtight when connected firmly.

### Contact With the Tubing System Does Not Trigger an Inflammatory Response

**Figure 3:**
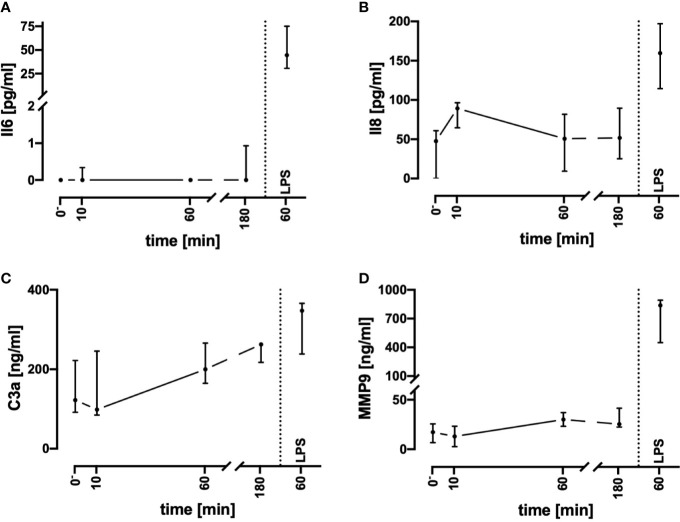
Assessment of humoral inflammatory activity during contact of whole blood with the tubing system by determining the plasma concentrations of Il6 **(A)**, Il8 **(B)**, C3a **(C)**, and MMP9 **(D)**. Incubation with LPS 100 ng/ml reflected the presence of PAMPs. n = 3 for 10 min and 180 min, n = 10 for all other time points. Results are presented as median with error bars indicating the interquartile range.

To assess inflammatory processes induced in the present model system, key inflammatory markers were determined (**Figure 3**). Of note, the plasma Il8, MMP9, and C3a concentrations remained in a stable range during the monitored incubation period of 3 h. In parallel, Il6 levels were mostly not detectable. Despite the complement system has not been broadly evaluated, it was unlikely that massive C5a generation occurred. It is established, that neutrophils respond on exposure to C5a with downregulation of CD88 ((31), **Figure 5B**). However, in the present model system, neutrophil CD88 expression was not significantly altered within the first hour of incubation, indicating excessive complement activity to be unlikely.

### Preservation of the Immune and Metabolic Response in the *Ex Vivo* Situation During the First Hour

**Figure 4:**
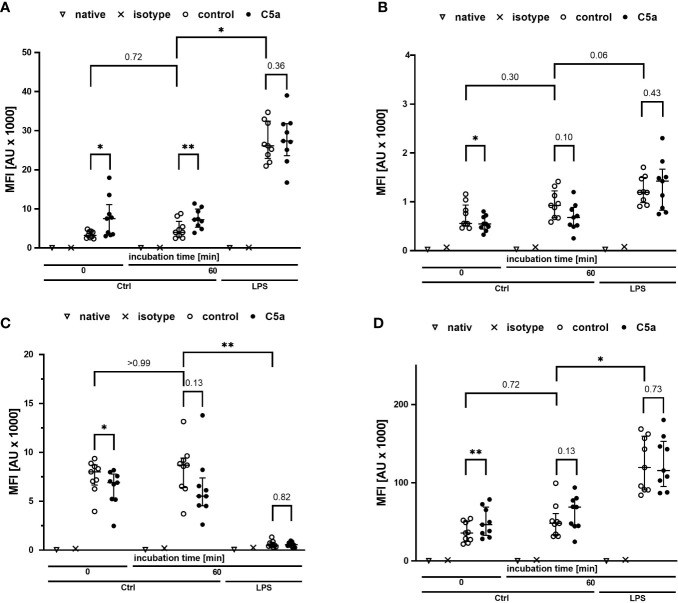
Profiling of activation markers CD11b **(A)**, CD88 **(B)**, CD62l **(C)**, CD14 **(D)** of monocytes directly after venipuncture (0) and after 60 min of incubation in the whole blood model with or without LPS (100 ng/ml). Following phlebotomy or after the given stimulation period *ex vivo*, monocytes were stimulated with C5a (100 ng/ml) *in vitro* for 15 min. n = 9, results are presented as scatter plot and median with error bars indicating interquartile range. * = *p* <0.05, ** = *p* <0.01.

**Figure 5:**
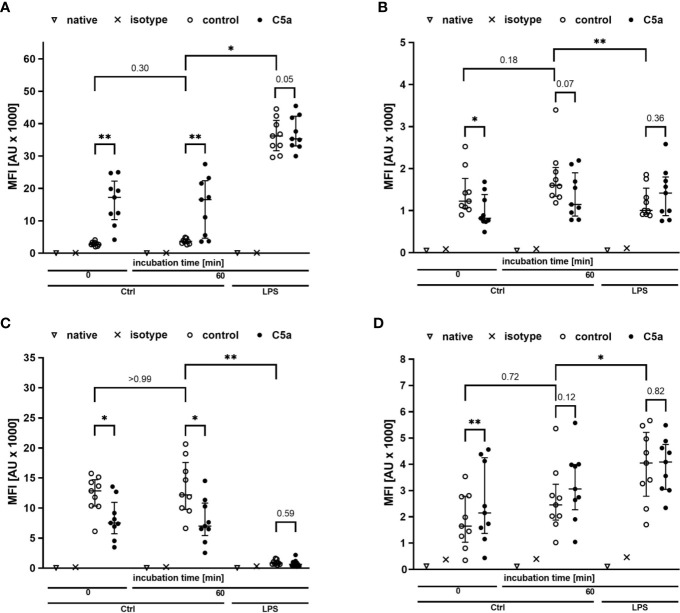
Profiling of the activation markers CD11b **(A)**, CD88 **(B)**, CD62l **(C)**, CD14 **(D)** of neutrophil granulocytes directly after venipuncture (0) and after 60 min of incubation in the whole blood model with or without LPS (100 ng/ml). Following phlebotomy or after the given stimulation period *ex vivo*, neutrophils were stimulated with C5a (100 ng/ml) *in vitro* for 15 min. n = 9, results are presented as scatter plot and median with error bars indicating the interquartile range. * = *p* <0.05, ** = *p* <0.01.

Monitoring of the activation of monocytes and neutrophils was performed by simultaneously analyzing the expression patterns of CD11b, CD88, CD621, and CD14 as well-established markers of the innate immune response after LPS exposure and/or during sepsis. **Figures 4**, **5** summarize the findings for the incubation period of 1 h, showing no significant alterations after contact within the whole blood model. Additionally, the cellular response pattern of monocytes and neutrophils to *in vitro* stimulation with C5a either directly after venipuncture or during a defined period in the whole blood model was multi-parametrically evaluated. For all activation markers, there was no relevant change in the cellular response of monocytes or neutrophils before or after incubation in the whole blood model after subsequent *in vitro* stimulation with C5a.

**Figure 6:**
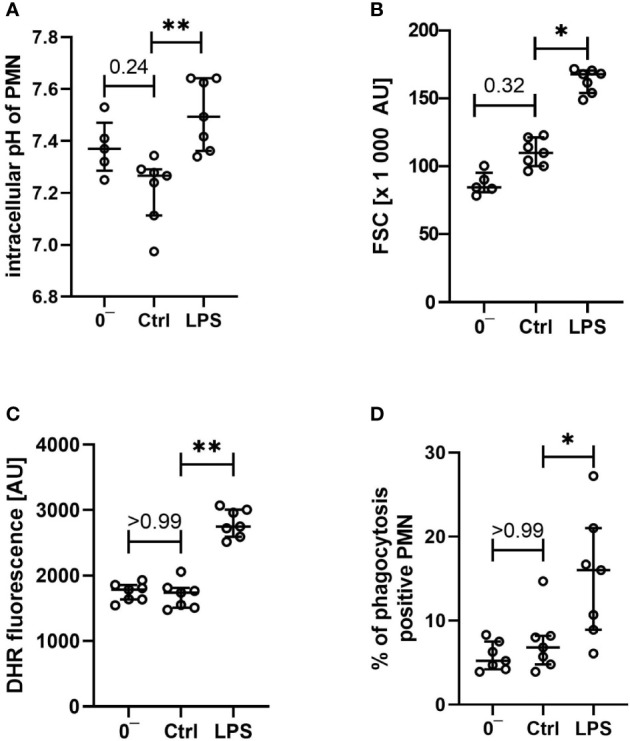
Characterization of intracellular pH **(A)**, size **(B)**, generation of reactive oxygen species **(C)**, and phagocytotic activity **(D)** of neutrophils after venipuncture (0^−^, n = 5 – 7) and after incubation in the whole blood model for 1 h with LPS 100 ng/ml or without further stimulation (Ctrl, both n = 7), results are presented as scatter plot and median with error bars indicating the interquartile range. * = *p* <0.05, ** = *p* <0.01.

In addition to the reported extracellular parameters, neutrophils were analyzed regarding the cellular physiology and functionality. As presented in **Figures 6A, B**, the intracellular pH and the cellular size of neutrophils were not changed after contact with the whole blood model, apart from a slight intracellular acidification being in accordance with the changes noted in the extracellular pH. Moreover, vital key functions of neutrophil granulocytes remained intact: ROS generation (**Figure 6C**) and phagocytotic activity (**Figure 6D**) remained largely stable when comparing neutrophils before and after exposure to the whole blood model, besides a small shift in intracellular pH and FSC.

### Stimulation With LPS Causes Sepsis-Like Changes of the Immunologic Phenotype *Ex Vivo*

LPS (100 ng/ml) was used as a well-described and potent activator of innate immunity, demonstrating that the whole blood model is fully capable of generating a proinflammatory immunologic phenotype. Incubation of the whole blood system with LPS for 1 h activated the cellular metabolism. Glucose utilization was significantly enhanced by 24% (15%; 49%; Ctrl: −13 mM/h; −16; −11; vs. LPS −17 mM/h; −20; −15; *p* < 0.01, n = 10). Similarly, lactate generation was significantly increased by 27% (22%; 33%; Ctrl: +1.3 mM/h; 1.1; 1.6; vs. LPS: +1.7 mM/h; 1.5; 1.9; *p* < 0.01, n = 10). Otherwise, global parameters of differential blood count and blood gas analysis remained widely unchanged. As a subsequent step, we compared the C3a, Il6, Il8, and MMP9 levels after stimulation of the whole blood *ex vivo* with LPS. LPS induced a tremendous increase in Il6 and MMP9, while Il8 and C3a were slightly increased (**Figure 3**). Interestingly, LPS-induced generation of MMP9 attained a maximum level within the first hour with a concentration that was 10–20-fold increased after 1 and 3 h of stimulation in comparison with the control specimens (data not shown). By contrast, LPS-induced generation of Il6 continued to increase beyond 1 h, resulting in a greatly higher level after 3 h (1 h: 45 pg/ml; 31; 75, vs. 3 h: 2523 pg/ml; 2271;2889, data not shown, n = 10 and n = 3, respectively).

On the cellular site, monocytes and neutrophils responded to LPS exposure with an increase in the expression rates of CD11 and CD14, while CD62l was markedly decreased. The expression of CD88 was increased in monocytes and decreased in neutrophils after exposure to LPS (**Figures 4**, **5**). Furthermore, the cellular response pattern to additional stimulation *in vitro* with C5a was significantly impaired. In addition to extracellular activation markers, LPS incubation in the whole blood model altered intracellular parameters of neutrophils, increasing both intracellular pH and size (**Figures 6A, B**). Moreover, the activity of neutrophils regarding ROS production and phagocytosis was significantly enhanced (**Figures 6C, D**).

## Discussion

### Blood Physiology and Inflammation in Context

**Figure 7:**
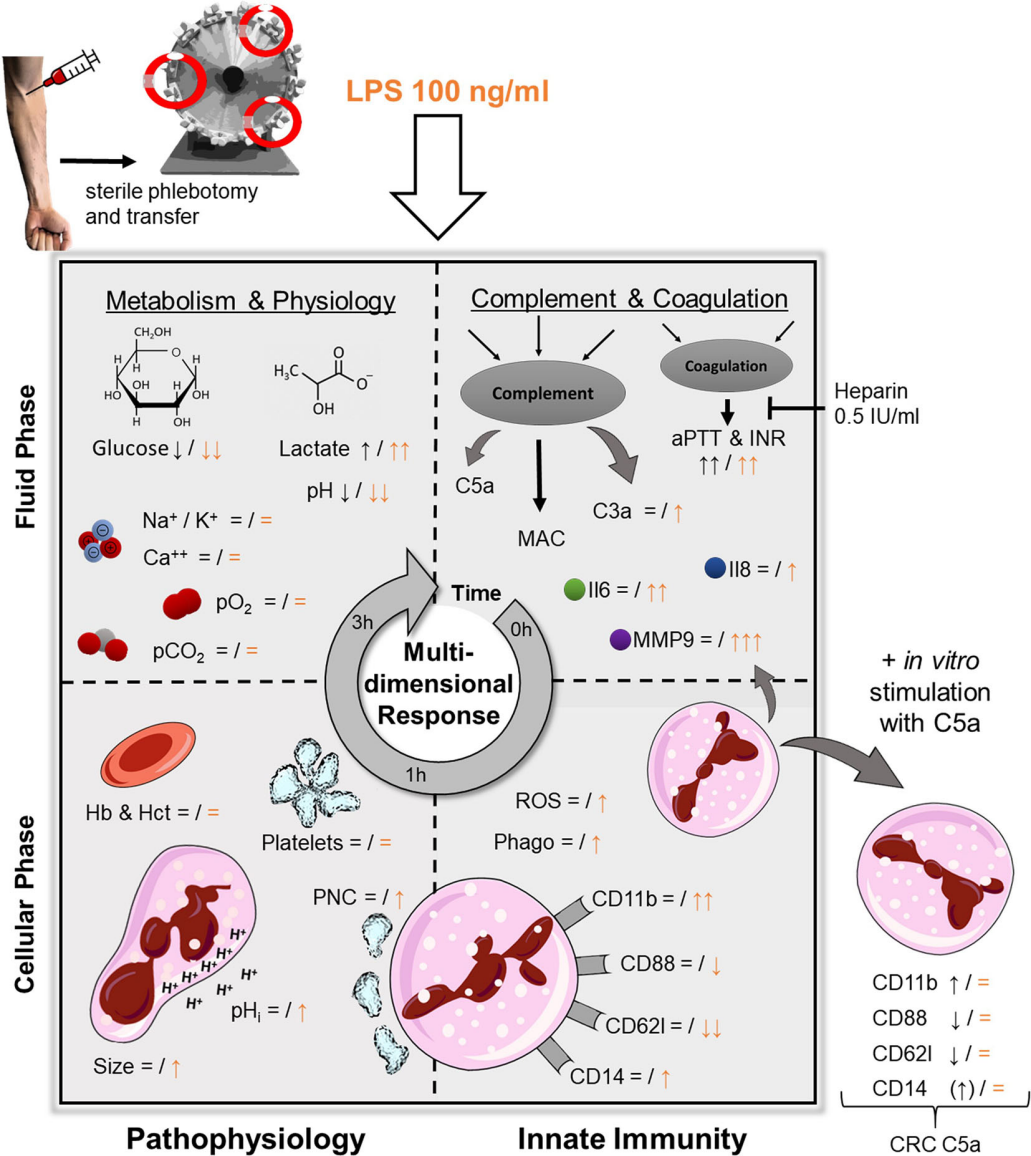
Graphical abstract of the *ex vivo* whole blood model and the LPS-induced simulation of sepsis. Black symbols represent changes after 1 h of contact with the tubing system, orange indicates a concomitant stimulation with LPS (100 ng/ml). pO_2_ and pCO_2_, partial pressure of oxygen and carbon dioxide, respectively; Hb, hemoglobin; Hct, hematocrit; PNC, platelet-neutrophil-complexes; pH_i_, intracellular pH; C3a and C5a, complement factor 3a and 5a, respectively; MAC, membrane attack complex (sC5b-9); aPTT & INR, activated partial thromboplastin time and international normalized ratio, respectively; Il, Interleukin; MMP9, matrix metallopeptidase 9; ROS, reactive oxygen species; Phago, phagocytosis; CRC, cellular response capacity (to subsequent additional stimulation with C5a.

In extracorporeal circulation, the blood system is subjected to various alterations because the interaction with other organ systems is interrupted while in parallel the continuous contact and interaction with the intact endothelium is lost. Furthermore, the *ex vivo* blood is exposed to artificial surfaces, resulting in activation of thromboinflammation (32). In the current model, the coagulation cascade was inhibited on the material surface and in the blood by a steady level of heparin, however, without administering an extensive amount. Of note, the platelet count and PNC formation remained stable (while still inducible by LPS), indicating no relevant activation of cellular coagulation. Nonetheless, one must consider, that heparins of both high and low molecular weight have been shown to directly interact with immune cells, for example, by increasing myeloperoxidase activity (25), although, a much lower concentration of heparin (0.5 IU/ml) was used in the present model. It is also noteworthy, that none of the activation markers assessed in this model were significantly elevated after 1 h (**Figure 7**). Regarding metabolic changes of blood physiology, alterations in blood pH, glucose levels, and lactate were noted in the current model. While acidification and lactate levels were tolerable within the first hour, they cannot be considered physiological after 3 h. In a similar manner, blood became hypoglycemic. Cellular innate immunity (particularly neutrophils) mainly relies on anaerobic glycolysis for its metabolism (33, 34). Furthermore, cellular effector functions, including phagocytosis, require anaerobic glycolysis (35). Also, hypoglycemia has been shown to aggravate the response to inflammatory stimuli, including LPS (36). In parallel, (lactate) acidosis modulates many activities of the immune system, for example, reducing the phagocytotic capability of neutrophils while increasing their production of MMP9 (37).

Increased Il6 and MMP9 levels are well described in inflammation and during sepsis (38, 39). The Il6 levels after 1 h of stimulation with LPS had almost attained the threshold level of septic patients (39, 40). In this regard, it is, however, likely that Il6 during sepsis *in realiter* can be present longer and attain higher concentrations, because normally more than 1 h elapses until the patient appears in the clinic with the diagnosis of sepsis. By contrast, the MMP9 levels in the model system were far above the concentrations described in sepsis (38) as early as 1 h after incubation. It is tempting to speculate that the potent interactions of MMP9 with the glycocalyx of the endothelium, particularly during systemic inflammations that result in degradation of the glycocalyx, which was of course absent in the current *ex vivo* simulation, may induce higher MMP9 levels *ex vivo* in comparison with the *in vivo* situation.

Overall, changes in cellular innate immunity closely resembled the described phenotype alterations in modeled systemic inflammation. For example, in two independent *in vivo* models of LPS administration in either rats or mice, neutrophil CD62l and CD11b expression appeared similar to the patterns found after LPS exposure in the *ex vivo* whole blood model (30, 41, 42). Reduced CD62l expression was also observed in neutrophils from patients with sepsis and endotoxin-challenged human volunteers (8, 43). Varying CD11b expression profiles of neutrophils have been reported in clinical sepsis (43, 44). These potential differences in CD11b expression (and the differences noted above regarding MMP9 and Il6) may either reflect some limitations of sepsis simulation by isolated LPS stimulation and/or the restricted observation period in the present model. However, the present results regarding the LPS-induced changes of neutrophil expression of CD11b, CD62l, and CD88 are perfectly in line with other whole blood models (45). Additionally, the reduced CD88 expression on leukocytes after simulation of inflammation in the whole blood model is corroborated by data from severely injured, sick, and septic patients (46–49).

In addition, LPS stimulation in the present model resulted in a similar shift of intracellular parameters in comparison with *in vivo* findings in recent literature. For example, an alkalization of neutrophils has been reported in septic shock patients (28). Likewise, a rise in size of granulocytes has been described in a murine model of cecum ligation and puncture-induced sepsis (27) and patients after trauma-induced inflammation (50).

### Whole Blood Models in Context

Addressing the complex pathophysiology of systemic inflammation and blood stream infections requires models that can sufficiently portray the various cross-talk mechanisms between the blood cells, the endothelium, the humoral agents of immunity and hemostasis as well as blood physiology in terms of thermoregulation, acid-base-homeostasis, and blood-gas characteristics. Therefore, a plethora of different approaches and protocols were developed to allow the *ex vivo* investigation of the complex and highly sensitive organ “blood”. In this context, the term “whole blood” mostly represents an approach whereby blood, which has been somehow anticoagulated, retains the functionality of most if not, in principle, all its cellular and humoral components. Some protocols apply anticoagulated blood for studies which require an intact immune system, including simulating blood-stream infections (16), analyzing vaccine safety (17), and investigating leukocyte trafficking (51). These models usually add heparin or a hirudin-derived anticoagulant to mainly preserve the complement system as an important calcium-dependent humoral component of innate immunity. By contrast, citrate or Ethylenediaminetetraacetic acid addition interferes with the complement system (32).

Blood models designed to investigate the coagulation cascade, particularly cellular hemostasis, normally use citrated blood including a protocol to resubstitute calcium in a stoichiometric manner; however, attempts have been undertaken with hirudin-based models (52). Other groups established a closed *in-vivo*-like setup by combining defined cell layers or cell culture-based organoids with whole blood (53, 54).

### *Ex Vivo* Incubation of Human Whole Blood—Advantages and Limitations

The presented animal-free *ex vivo* whole blood model has various advantages: First, it is effortlessly transferable, widely accessible, and convenient to set up, because all the components are commercially available and can be delivered under sterile conditions. Moreover, the materials do not require any special treatment, for example, coating by the user, thus improving standardization and reducing the risk of any contamination. Second, particularly within the first hour, whole blood incubated in the circuit system remains stable regarding both blood physiology and cellular innate immunity. Third, the model allows incubation of several milliliters of blood, enabling the collection of a sufficient amount of specimen for endpoint analysis. The application of an *ex vivo* stimulation in general allows inclusion of older, frailer individuals, because they are generally underrepresented in inflammatory studies, especially in *in vivo* LPS challenges (9). Fourth, mimicking the inflammatory stimulus of sepsis with LPS resulted in an immunologic phenotype in accordance with preexisting literature. Last and most importantly, such a reliable model may help support disseminating the 3R-principles of animal research (7) by offering a valid reduction and/or replacement strategy for the investigation of blood-borne and blood-transferred inflammatory and infectious responses.

There are also some limitations of the model. One restriction is the requirement of anticoagulation to reduce the activation of the blood while drawing and transferring it as well as presumably because of the shedding of at least some of the heparin coating into the system. Other whole blood models use different anticoagulants other than heparin, for example, hirudin, which inhibits the coagulation cascade further downstream than heparin. However, because of to the costs of hirudin-based drugs (e.g. lepirudin), and to adhere to the same substance as used for the coating of the tubing, we decided to apply a minimal dose of heparin systemically in the present model. Furthermore, the circulation of the continuously rotating air bubble only partially imitates the circulation forces in the human body without imitating the heterogeneous changes in pO_2_, CO_2_, or flow kinetics in the arterial and venous phases of circulation. Therefore, the changes in pO_2_ and the stability of CO_2_ are because of gas exchange with the remaining air bubble in the system, maintaining stable blood gas levels and being comparable to the venous environment. Another limitation is that the simulation of systemic inflammation occurs outside the human body, thereby excluding the blood-organ crosstalk, particularly the interactions with the endothelium, bone marrow, liver, and spleen. However, the current *ex vivo* setting allows the examination of inflammatory processes uniquely occurring in the blood itself. Finally, the timeframe for analyzing “physiologic” blood *ex vivo* is limited, because, for example, blood glucose is depleted and metabolic byproducts like lactate are not cleared. However, LPS-induced changes in the phenotype of neutrophils is a rather fast process, that starts within minutes and reached its ceiling as measured by CD62l and CD11b expression on neutrophils within 1 h (30). Future development might overcome these issues, for example, by supplementing glucose during the experimental course, by connecting the system to a pulsatile pump allowing the circulation of the blood through various organoids such as liver or kidney cells. In addition, although the model in its current setup is easy to perform, a desirable automatization process of the blood handling would be rather complex to achieve.

Overall, based on the data obtained from the synchronous monitoring of a comprehensive arsenal of physiological, metabolic, and immunologic blood parameters in an animal-free environment, we propose an *ex vivo* whole blood model of sepsis as a valuable addition to *in vivo* and clinical studies. Further studies need to validate the advantages of this model system in other inflammatory conditions and with blood from patients, for example, to test immunomodulatory treatments in blood from patients with systemic inflammation, cancer, or other diseases without putting patients at risk. Also, this model can be used as a screening tool, which may help to further reduce and/or replace animal experiments.

## Data Availability Statement

The raw data supporting the conclusions of this article will be made available by the authors, without undue reservation.

## Ethics Statement

The studies involving human participants were reviewed and approved by: Local Independent Ethics Committee of the University of Ulm. The participants provided their written informed consent to participate in this study.

## Author Contributions

Conceptualization and supervision: DM and MH-L. Data curation and formal analysis: DM, LV, ME, AS, JB, SH, and MH-L. Methodology: DM, CB, AA, KN, BN, and MH-L. Validation and visualization: DM, LV, and ME. Writing—original draft: DM, CB, and MH-L. Writing—reviewing and editing. All authors contributed to the article and approved the submitted version.

## Funding

The present work was funded by a research grant (“Forum Gesundheitsstandort”) of the Ministry of Science, Research, and Art Baden Wuerttemberg to DM and MH-L, a start-up grant and a position as Clinician Scientist to DM by the Collaborative Research Center 1149, project number 251293561, German Research Foundation, as well as grants to KN and BN (grants 2018- 04199, 2016-2075-5.1, and 2016-04519) provided by the Swedish Research Council. The funders had no role in the design of this study, data collection and interpretation, or decision to submit results.

## Conflict of Interest

The authors declare that the research was conducted in the absence of any commercial or financial relationships that could be construed as a potential conflict of interest.
